# Towards Controlled Single-Molecule Manipulation Using “Real-Time” Molecular Dynamics Simulation: A GPU Implementation

**DOI:** 10.3390/mi9060270

**Published:** 2018-05-29

**Authors:** Dyon van Vreumingen, Sumit Tewari, Fons Verbeek, Jan M. van Ruitenbeek

**Affiliations:** 1 Huygens-Kamerlingh Onnes Laboratorium, Universiteit Leiden, 2333CA Leiden, The Netherlands; dyon@vanvreumingen.nl (D.v.V.); mecsumit@gmail.com (S.T.); 2 Leiden Insitute of Advanced Computer Science, Universiteit Leiden, 2333CA Leiden, The Netherlands; f.j.verbeek@liacs.leidenuniv.nl

**Keywords:** graphics processing unit, single-molecules, controlled manipulation, scanning tunneling microscope, molecular electronics, real-time simulation, parallel computing

## Abstract

Molecular electronics saw its birth with the idea to build electronic circuitry with single molecules as individual components. Even though commercial applications are still modest, it has served an important part in the study of fundamental physics at the scale of single atoms and molecules. It is now a routine procedure in many research groups around the world to connect a single molecule between two metallic leads. What is unknown is the nature of this coupling between the molecule and the leads. We have demonstrated recently (Tewari, 2018, Ph.D. Thesis) our new setup based on a scanning tunneling microscope, which can be used to controllably manipulate single molecules and atomic chains. In this article, we will present the extension of our molecular dynamic simulator attached to this system for the manipulation of single molecules in real time using a graphics processing unit (GPU). This will not only aid in controlled lift-off of single molecules, but will also provide details about changes in the molecular conformations during the manipulation. This information could serve as important input for theoretical models and for bridging the gap between the theory and experiments.

## 1. Introduction

Researchers have been interested in understanding electronic transport through single molecules for several decades. Small molecules due to the confinement of electrons exhibit large energy level spacing and can thus show quantum dot characteristics even at room temperature. Single molecules can show conformational variations [1], which could be used to make a switch, as these different conformations can vary significantly in their conductance. Quantum interference [2,3], thermo-electricity [4,5] and molecular spintronics [6,7] are some of the many exotic phenomena that one can study in single molecules. There are many things we have learned so far, but many are still to be understood and put under control from both the fundamental and technological point of view. One of these things is the coupling between the molecules and the leads at the atomic scale. In particular, the atomic scale structure of the contacts and the details of the binding of the molecule to these leads have to be known. These details include knowledge of the binding sites, the nature of the bond, the conformational state of the molecule, the bond angles, etc. These are important parameters to know because our understanding of electronic transport through single molecules depends on the output of various computational models like density functional theory (DFT), non-equilibrium Greens function (NEGF) theory, etc., which require these parameters as inputs. Lack of such information can cause a mismatch between the theory and experiments, which affects our understanding of electron transport through single molecules.

Furthermore, one can ask: Is it possible to set some benchmark experiments that could help in testing different computational models and also enhance our understanding of electron transport through single molecules? Most commonly-used single-molecule measurement techniques include: various break junction techniques (notched-wire [8,9,10], lithographic [11,12,13], scanning tunneling microscope [14] based), electromigration (metallic) [15,16] and electroburning (graphene) [17,18] junctions. The break-junction techniques rely on doing statistics on a large dataset, thus averaging over many molecule-electrode configurations during trace-retrace cycles where the two electrodes are brought into large metallic contact and subsequently broken. These techniques thus inherently cannot provide information about the exact atomic configuration of the junction, which will be required for performing detailed atomistic calculations to determine coupling strength, conductance, etc. For this, we need to turn towards more single-shot measurements.

### Single-Shot Measurements

Scanning tunneling microscope (STM) owing to its nano-scale imaging possibilities has also been used to probe single molecules for single-shot measurement. If a molecule is attached between a tip and a surface in an STM, then the atomic configuration of one electrode (the surface) is precisely known. Note that this is not the case when STM is used in break-junction mode.

First, such a controlled single-molecule measurement where the STM surface atomic structure was intact was reported in 1995 by Joachim et al. [19], where they connected a single C_60_ molecule between the STM tip and an atomically flat Au(110) surface at 300 K under ultra-high vacuum (UHV) conditions. Later, single planar molecules connected between the STM tip and sample while keeping the atomic structure of surface intact were studied using I(t) and I(s)measurements reported by Haiss et al. [20,21] in ambient conditions. However, these measurements also require doing statistics on the current jumps that are detected as molecules spontaneously make and break contact between the tip and the surface when the STM tip is brought close to the flat metallic surface (with a low coverage of molecules). The first controlled peel off of a single planar molecule was performed by Temirov et al. [22] in 2008, where they showed successful removal of a PTCDA (perylenetetracarboxylic dianhydride) molecule from a Ag(111) surface using a W-tip. Even though PTCDA has an axially-rigid structure, variations in the behavior of the conductance traces were observed while peeling off the molecule. These variations could be attributed to different conformational changes that the molecule undergoes during the peel off or the changes in the binding sites of the molecule to the electrodes. Recently, a simple conductance measurement for a free-standing tripod molecule showed an order of magnitude variation [23] between that Au(111) surface and the Au STM tip depending on the different bond formation configurations between the tip and the molecule. This work again points towards the need to have a well-defined contact geometry of a metal-molecule-metal junction.

We suggest here a way to provide an “eye” to the STM during the manipulation process. Although this cannot replace experimental observations, it can serve as a tool for guiding the experiments. For this, we developed a system [24,25] where we attach a real-time molecular dynamics simulation to a low-temperature UHV STM. This simulation first takes the STM image as an input to create a 3D simulation stage similar to the experiment. Next, the same x,y,z signals are sent to both the STM and the simulation as an external user-input. The simulation computes in “real time” (this does not require simulations to be performed in native time-scales of the molecules) the dynamics of each atom in the system and provides a visual feedback to the operator, who can then choose an adaptable trajectory while monitoring its effect in real time on a computer screen. We also attached, for the ease of making adaptable 3D trajectories, a home-built 3D motion control sensor similar to Green et al. [26]. The real-time simulation employed for the experiments was applied for only metallic systems with a gold STM tip, a Au(111) metallic surface and with gold ad-atoms. Using this, the controlled creation and lift-off of a chain of gold ad-atoms over an atomically flat Au(111) surface were demonstrated. In this article, we will describe an extension of this real-time molecular dynamics simulation to include organic molecules. Due to the higher vibrational frequencies of the atoms in the molecule, we need to reduce the time step in the calculations, which slows it down considerably. In order to amend this problem, we prepared the simulation on a graphics processing unit (GPU) of the Nvidia architecture, using the CUDA application programming interface (API). GPU implementations of molecular dynamics have been demonstrated earlier. However, real-time simulations to control molecular manipulation using STM have not yet been demonstrated. In the following section, we will start by discussing some basics of molecular dynamics (MD) and GPU programming and mention the previous implementations of MD using GPU programming. Next, we will discuss the challenges involved in making this simulation real time and then present the solutions we provided, explaining the assumptions and improvements we made.

## 2. Molecular Dynamics

**Figure 1:**
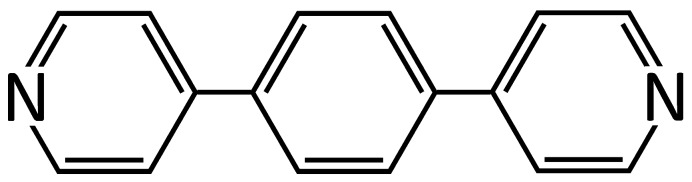
1,4-bis(4-pyridyl)benzene implemented in the simulation.

Molecular dynamics (MD) simulations have been used widely to understand bulk mechanical properties of materials arising from collective atomic behavior. The MD simulation presented here is intended to study and aid single-molecule manipulation experiments done using scanning probe microscopes. Our system thus consists of metal atoms, which form the tip and the sample used in the experiment, and other atoms (H, C, N, etc.), which constitute the molecule. The molecule that we studied to test our simulation is 1,4-bis(4-pyridyl)benzene, shown in Figure 1. The gold-gold interaction is modeled as before [24,25] using a semi-empirical potential. The atoms making up the molecule are joined together by covalent bonds for the chosen molecule and are thus modeled as spring-mass systems using harmonic potentials. Other than pair forces, the molecule’s atoms also feel angular forces due to equilibrium bond angles between two covalent bonds formed between three atoms and torsional forces due to equilibrium dihedral angles between a group of four atoms. All these bond pair forces, angular forces and torsional forces are computed to obtain the details of the molecular conformations. Lastly, the interaction between the atoms on the molecule and the gold atoms in the substrate and tip is modeled depending on the type of molecule atom. Atoms like N and S are known to form a stronger bond than H and C with gold and are thus called anchoring bonds. These anchoring bonds are modeled here using a Morse potential, while other gold-molecule bonds were modeled using the standard Lennard–Jones potential. A description of this is given in Appendix B.

The force calculations mentioned above have to be repeated for each atom. At each moment in time, these forces are all treated as functions of the inter-atomic positions of each atom. Thus, for a given position of all atoms, these calculations are all mutually independent and can be performed simultaneously, making the calculation suitable for parallel processing. We will discuss in the coming subsection the challenges in making a real-time simulation, and then, we will discuss our implementation using GPU programming, which can perform parallel processing of a large part of the computation.

### Challenges in Making Real-Time MD Simulations

There is always a finite number of atoms (*N*) simulated in molecular dynamics (MD) calculations, and the computation time of a classical MD simulation scales as $N^{2}$; this means that the need to crank up the simulation speed is always there as one can then simulate more atoms at a time. The time-scales involved with inter-molecular motions are on the order of femtoseconds [27]. This is why speeding up the simulation to calculate inter-molecular motions and vibrations in their native time-scales is not possible with the current computing power. This implies that a real-time MD simulation should not be confused with native time-scale (NTS) computations. However, real-time molecular dynamics can be performed on any system in the quasi-static regime. The quasi-static regime is where the relaxation time (the time it takes for the atoms in the system to relax to the equilibrium position within a certain accuracy) in the simulation, which will depend on the computation speed of our system, is smaller than the pump and probe period.

The primary challenge that we face here is due to the very small masses of the atoms constituting the molecules (like H, C, N, etc.) in comparison to the mass of the Au atoms, which form the tip and the substrate. From Newton’s second law, it can be seen that a smaller mass will lead to much higher acceleration under the same force, and thus, it is required to use a much smaller time step in the simulation. The larger time step applicable for the gold-gold system will not work here, as it will lead to stretching of bonds between smaller atomic masses, and thus, can lead to spurious decomposition of the molecule in the simulation. Using a smaller time-scale optimized for the smaller mass should ideally work for all the masses. However, it will lead to two problems in our situation. Firstly, this time-scale has to be larger than the one associated time-scale of the external impulse used to steer the STM tip [24,25]. Otherwise, the Au atoms in the tip being heavier will not follow the external steering input coming from the 3D motion control system. Secondly, if we make the external steering too slow, then the simulation will not work on the time-scale of the real STM manipulation experiment.

GPU programming will help in increasing the computational speed, but will not be enough to achieve a real-time implementation when using the smallest single time step. We have solved this time step problem using a multiple time step technique similar to what was proposed by Tuckerman et al. [28] in 1991. A discussion about it will follow below.

## 3. Molecular Dynamics: GPU Implementation

As was explained earlier in the Molecular Dynamics Section, the bulk of the execution time in MD simulations is consumed by routines calculating the inter-atomic forces, which do not in any way depend on one another. After all, these forces are derived from potentials that are functions only of atom positions. Therefore, such mutually independent calculations can easily be formulated in a parallel computing format. In fact, there are even dedicated special purpose parallel computing systems built for molecular dynamics simulations called MDGRAPE made by RIKEN research institute in Japan. This machine is used to study protein folding predictions [29]. The latest MDGRAPE-3 is a supercomputer with a 947-TFLOPS (terraFLOPS) level of floating point performance. Other than this specialized hardware, many GPU-based implementations [30,31,32] of molecular dynamics have also been demonstrated.

GPUs were originally designed to do specific tasks of graphics processing, but soon, it became obvious that making these GPUs do more versatile jobs and handle different types of functions would enhance their potential. It was this observation that led to the introduction of general purpose GPU (GPGPU) programming, which found its applications in seismic, weather and medical research, in neural networks and atomic simulations. An important advantage of GPGPU is that it allows data or information transfer in both directions, from CPU to GPU and GPU to CPU. We used the compute unified device architecture (CUDA) API as our GPGPU programming interface and an Nvidia GeForce GTX 960 graphics card (cf. Table 1).

**Figure 2:**
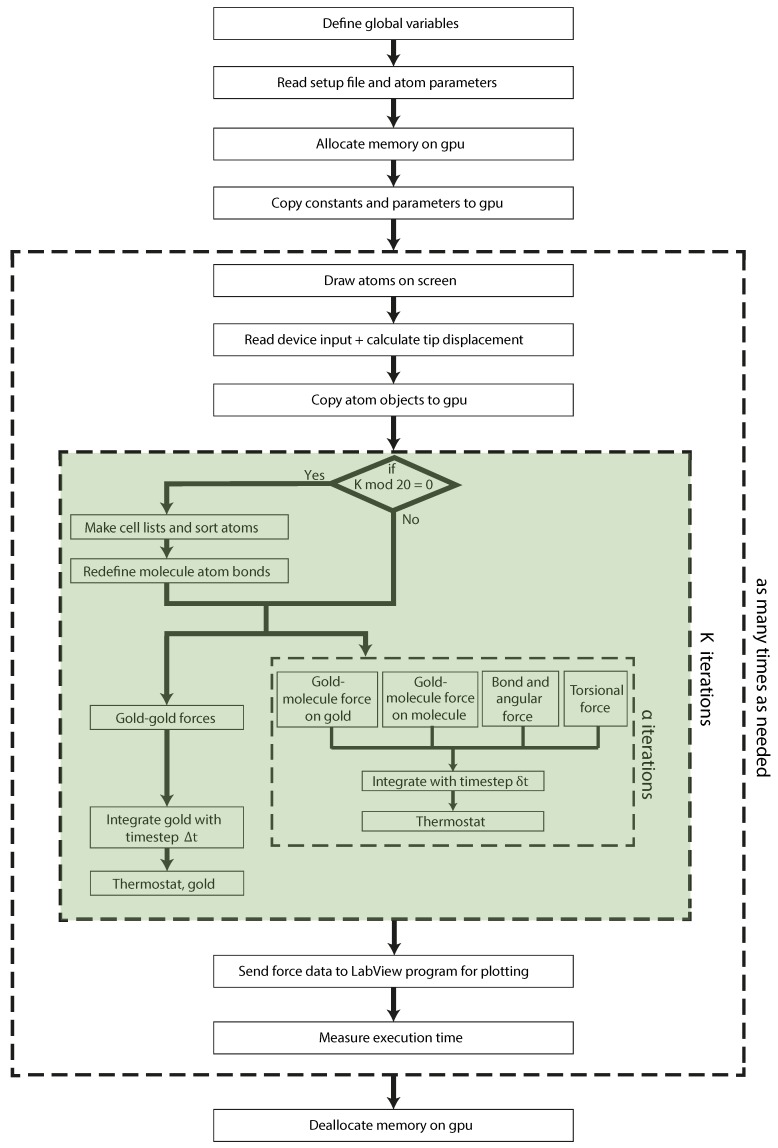
Flowchart showing the execution scheme of the program. Time runs in the vertical direction; thus, subroutines placed next to each other run in parallel. Parts of the chart contained in dashed boxes run as many times as stated to the right of the boxes, respectively.

The benefit of implementing a molecular dynamics simulation on a graphics card lies in the high degree of parallelism that can be achieved when many independent tasks are executed simultaneously. However, not every component is suitable for running on a GPU: while the graphics chip may contain much more processing cores than the CPU, they run at a lower clock speed and lack an interface for correspondence with any other devices. Therefore, we carefully selected the parts that were most suitable for being computed on a GPU, which are the force calculations, velocity Verlet integration and the thermostat. The elements then remaining on the CPU include handling the data from the input apparatus and moving any recorded force values to a small LabView graphing program for real-time plotting (if needed). Plotting and controlling the visualization of the atoms on the screen were done on an integrated graphics chip, using a graphics library (https://www.sfml-dev.org/), which has been implemented in terms of CPU-callable methods. Figure 2 shows a flowchart that includes all core tasks performed by the program. The parts implemented inside the GPU are shown within the green box. The time runs along the vertical axis, and the execution of the processes is in the order from top to bottom. We will now go through the details of the kernel implementations, which distribute the tasks among different thread blocks.

### 3.1. Gold-Gold Forces

**Figure 3:**
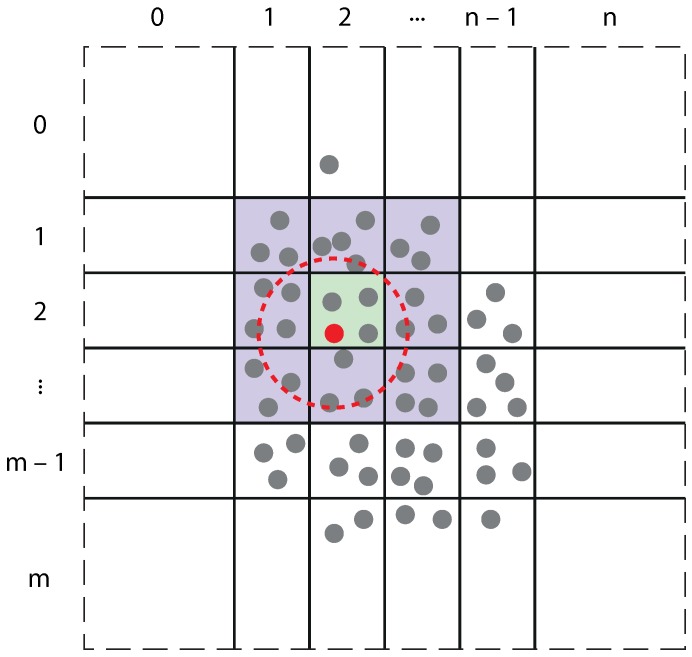
Gold substrate divided into cells. For the atom colored in red, the cut-off radius is indicated as a red, dashed circle, while its own cell is depicted in green and its neighboring cells in blue. Atoms whose positions are outside the cut-off sphere of the indicated atom are not taken into account in calculating the total force on the said atom. Note that the outermost cells have no boundary; this measure was taken to fit all of the 3D space (an atom may end up anywhere, after all) into a finite number of cells.

The algorithm that we use for calculating the interaction forces between the gold atoms in the substrate and the tip is based on the cell list method [33]. This approach differs from other methods such as the neighbor list method [34] where each atom is assigned its unique index. In the cell list method, each atom is placed in one of many cells that together make up the whole structure. In our implementation, the dimensions of these cells are set to be equal to a cut-off radius. This cut-off radius is normally defined to speed up an MD simulation [27], where the forces acting on an atom are only computed from atoms lying within this cut-off radius, assuming the inter-atomic interactions are negligible beyond this cut-off radius. In this way, we can guarantee that all neighboring atoms that are located within the cut-off radius of atom *i* must lie in one of the cells directly neighboring the cell that atom *i* was placed in; see Figure 3. This method has several significant advantages: firstly, it imposes a data structure on the atom objects according to their position, something that is not directly achievable with a simple atom list. Secondly, since we know that all atoms interacting with atom *i* must be in one of the neighboring cells (due to the cut-off radius), only a few instead of all distances between atoms now have to be calculated. Thus, the number of pair distances to be computed in each run becomes independent of the size of the system, and therefore, it reduces the complexity of the algorithm from O($N^{2}$) to O(*N*). For the ease of implementation, we choose a two-dimensional grid structure for these cells. Here, in the third dimension (*z*-direction), all cells stretch from negative infinity to positive infinity.

Now, from the GPU memory model side, each atom cell is assigned to one thread block where the computation of gold-gold forces on each atom is assigned to one thread inside the thread block. The threads in the thread block are arranged in a one-dimensional order with an individual index (say $\left(\tau_{1},0,0\right)$) for each thread. A cell count list is also prepared to keep track of the number of atoms in a cell. This helps the kernel to command each thread to loop over all the neighboring atoms and calculate the forces within the cut-off radius (Notice that if the number of gold atoms is less, then to increase the occupancy of threads, we could use a higher dimensional thread block with indices $\left(\tau_{1},\tau_{2},\tau_{3}\right)$, thus also avoiding the for-loop. However, this is not essential at this stage, as we have a sufficiently large number of gold atoms to be computed.). These forces are summed and integrated over a time step Δt. As the kernel can only assign a task to the threads in the group of 32 given by the warp-size, so the number of atoms in a cell shown in Figure 3 has to be a multiple of 32. The warp-size of 32 is set by Nvidia, and it reserves the right to change this in the future. The larger the number of atoms we will have in the cell, it will require each thread (which is assigned per atom) to iterate over a larger number of neighboring atoms (including those in the neighboring cells), which will make it computationally expensive. Second, each thread block (computing over each cell) needs to store the positions of all the atoms in the cell and in all the neighboring cells, as well. That means a larger number of atoms in a cell will require larger shared memory usage. The above two reasons make it imperative to restrict the number of atoms that can fit in a cell to 32 for the simulation to work optimally. Thus, any atom exceeding this limit is not taken into account in the force calculation. Because of this, the substrate may consist of only a few layers, depending on the cut-off radius (for a larger substrate or for a molecule, a 3D cell structure can be used).

It is important to note that threads in each thread block can only access information between each other using the shared memory and not among other thread blocks (or other cells in our case). Thus, it makes sense to have the threads in each block first load all of these positions (of atoms in a cell and its neighbor cells) into shared memory, before checking whether the distances fall within the cut-off radius and eventually calculating the forces. Note that this would not have worked out without the cell structure since the atom positions of the whole substrate would require such a large chunk of shared memory, that this would severely limit the number of blocks that could run on the GPU at the same time. Thus, the cell structure offers a nice way to make use of thread blocks and, more importantly, shared block memory. It goes without saying that all the functions accounting for this data structure management can run for all atoms in parallel, which ensures that the caused overhead stays small relative to the total force computation time. It turned out that refreshing the cell index and cell count lists only once every 20 iterations had barely any effect on the performance of the simulation; thus, we chose not to call this routine every iteration.

### 3.2. Forces on Molecule Atoms

The gold-molecule forces on molecule atoms and the intramolecular forces within the molecule are essential as they are responsible for the manipulation of the molecule over and out of the surface. As mentioned earlier due to the small masses of the atoms constituting the molecule, the equations of motion calculations cannot be done with the same time step as for the gold atoms, and thus, we need to choose another time step for the molecule. However, this will give an inconsistency in the interface at the junction between gold and molecule. To solve this issue, we use a method similar to what was proposed by Tuckerman et al. [28]. They have shown that for a system with heavy and light particles that requires different time steps, one can keep the background of heavy particles fixed to their positions, while light particles’ forces and positions are being computed using the smaller time steps. The larger particles are then computed and updated only in the larger time steps. They further show that for systems with a large number of heavy particles and a small number of light particles, the above multiple time step technique can also provide a huge speed-up in the computation.

Following this idea, we decided to compute the gold-molecule forces and the intramolecular forces with a smaller time step (δt). Moreover, for every α(∝Δt/δt) iterations of small time steps computed, one iteration of gold-gold interactions updating the positions of gold atoms is done using a larger time step (Δt) as described in the previous section. Now, to compute these gold-molecule forces and also intramolecular forces, two-dimensional thread blocks are employed. This is useful for increasing the thread occupancy of the GPU as the number of molecule atoms is not many. To avoid data race conditions (data races are a common problem in parallel programming and happen when two threads try to change some value in the same memory address: both read and change the value at the same time, which causes the returned value of the fastest thread to be overwritten by the other thread, thus giving the wrong result) while computing and summing the net force on each atom in a parallel manner, the so-called parallel reduction method [35] is used. The memory allocations for gold-molecule forces and intramolecular forces are done differently from each other in their respective kernels, as is discussed below.

#### 3.2.1. Gold-Molecule Forces

It is important to understand the computational difficulties that arise at the gold-molecule interface. An interface gold atom (let us say the tip apex atom, which is in contact with the molecule) is affected both by the external input to the tip and the molecule relaxation. This complicates the parallel division of the computation tasks. For an efficient division of tasks among the two parallel computations, we divide the system into two parts: first, an only gold-atoms sub-system and second an extended molecule sub-system (i.e., a system with the molecule including the interface gold atoms). In the simulation, first the positions of interface gold atoms are computed solving the first sub-system using the large time steps (Δt) followed by readjusting its position doing α iterations with the small time steps (δt) for the extended molecule sub-system. Parallelism is exploited here by performing again a single iteration for the first sub-system while these α iterations are performed, thus computing the next position of interface gold atoms based on the first sub-system calculation. At the end of one of such k-iterations’ cycle (shown in Figure 2), the results for the positions for the interface gold atoms in the two sub-systems are added. Then, the system waits for the next external force input and starts with the whole cycle again.

For its implementation in the GPU, we introduce here another cell index, which makes it easy to differentiate between molecule and gold atoms in the same cell. Then, similar to the gold-gold interaction case, for any molecule atom, the gold atoms that are close enough are identified for the force computation by looking at the neighboring cell indices. Then, a two-dimensional thread block is launched with size 32×27×1. The 32 columns in it correspond to the 32 atoms per cell, while each column is assigned 27 threads, which are taken because it is the largest multiple of nine (the number of cells that need to be considered for each atom) smaller than 32; thus, bringing the number of threads per block as close as possible to the limit of the Nvidia Maxwell architecture, which is 1024=32×32. Therefore, in comparison to the gold-gold interaction case where we had one thread per atom in the cell, now we have 27 threads per atom in the cell. These 27 threads help in computing the forces from the neighboring gold atoms in parallel. Depending on the chosen cut-off radius, it might be the case that the number of neighboring gold atoms is more than 27 and thus each of these 27 threads might also have to run a small for-loop over a few neighboring atoms’ indices.

#### 3.2.2. Intramolecular Forces

The intramolecular force calculations involve only forces between the molecule atoms, and all the external forces from surrounding gold atoms calculated above will be added later. Since we have only molecule atoms connected to each other by covalent bonds, we can have a more simplified cell structure. Here, all molecule atoms have up to three references to three neighboring molecule atoms. The intramolecular forces consist of bond forces, angular forces and torsional forces. For a molecule, with maximum *n* bonds per atom (here, single, double or triple covalent bonds are all counted as one bond), the maximum number of bond force calculations $n_{b}$ per atom is *n*. Next, the maximum number of angular force calculations $n_{a}$ per atom is $\frac{3}{2}n\left(n-1\right)$, and the maximum number of torsional force calculations $n_{t}$ per atom is $2n{\left(n-1\right)}^{2}$. A derivation of these maximum limits is given in Appendix A. Similar to gold-molecule forces to implement these force calculations using a GPU, we utilize a two-dimensional thread block with each column in this case describing the different intramolecular forces on each molecule atom. The maximum number of force calculations that can be done simultaneously for each atom is decided by the number of threads per column, which similar to before, cannot be larger than 32 per thread block.

For a maximum number of bonds per atom n=4, the molecule can be non-planar (example: tetrahedral geometry). Although, for this, the maximum number of bond pairs calculations ($n_{b}=4$) and the maximum number of angular force calculations ($n_{a}=18$) are well within the 32 number limit, but the maximum number of torsional force calculations ($n_{t}=72$) is much larger than 32. This could still be computed by using three thread blocks and/or pre-calculating the groups for a specific molecule. However, for this first implementation, we limit ourselves to molecules with n=3, i.e., planar molecules. This makes the maximum number of bond pairs calculations $n_{b}=3$, the maximum number of angular force calculations $n_{a}=9$ and the maximum number of torsional force calculations $n_{t}=24$. Hence, one thread block is still not large enough, and we use two thread blocks, one for calculating bond pairs and angular forces per atom and the other for doing torsional force calculations for each atom.

All these intramolecular forces and forces due to gold atoms on each molecule atom are added and then integrated with the smaller time step δt. For simplicity, a thermostat consisting of a constant friction factor is incorporated in the simulation to dissipate the excess energy pumped into the system from the hand-controlled tip movement. Depending on the choice of the substrate material and the molecule, one may have to test suitable thermostats.

## 4. Results and Discussion

**Figure 4:**
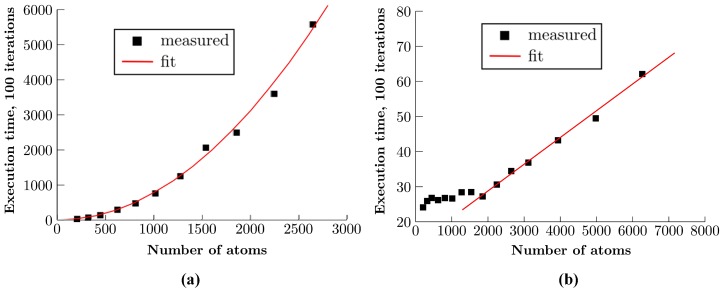
Total execution time, in milliseconds, for 100 iterations of atom relaxation with only gold interaction and motion enabled. (**a**) The graph shows the performance of the old CPU simulation, while (**b**) shows the calculation speed of our GPU simulation. In addition to the notable difference between the two implementations, we see that the time it takes the GPU to execute 100 iterations is nearly constant for a small number (less than 2000) of atoms, while a linear relation is observed in the cases with a higher number of atoms.

This GPU implementation allowed us to extend our previous all-metallic real-time simulation by including an organic molecule. In this implementation, all the intramolecular forces, i.e., bond forces, angular forces and torsional forces are computed. Along with this, the force that the metal atoms within a pre-defined cut-off radius (in STM tip and substrate) apply on each molecule atom was also computed. All these force calculations allowed us to keep track of the conformation changes that accompany a molecular manipulation operation. To test the performance of this GPU implementation, we successively increased the size of the substrate on which the simulation is performed, and we compare in Figure 4 the total time that was required to execute one hundred iterations on both the CPU and GPU. We can immediately see a striking difference in performance between the two: where the execution time of the CPU implementation quadratically increased and quickly reached the order of seconds, the GPU computation increased linearly and stays under 100 milliseconds even at a much higher number of gold atoms. Note that the linear dependence of the execution time on the number of atoms, which is inherent to the use of a cell list data structure, only appeared to hold from about 2000 atoms onwards. A possible explanation for this is that the GPU was not yet filled before the end of an iteration and could have taken on more threads. In other words, for a smaller numbers of atoms, the latency caused during synchronization with the CPU was limiting the execution time. The system we studied here contained only gold interaction, and we consequently increased the size of the gold substrate during the performance test. As the number of atoms in the molecule cannot be increased in the current GPU implementation due to the limit of 32 atoms per cell imposed by the warp size, in order to test the performance of our system, we increased only the number of gold atoms.

**Figure 5:**
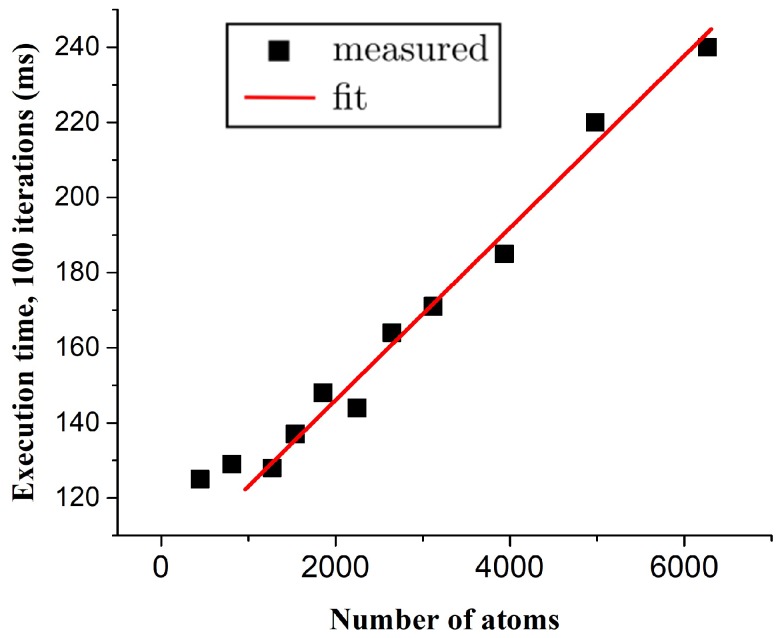
Total execution time, in milliseconds, for 100 iterations of atom relaxation including both gold and molecule computations. A linear relation between the substrate size and the execution time is observed. Again for a smaller number of substrate atoms, the execution time is nearly constant indicating an incomplete utilization of all GPU threads.

A similar performance analysis as before, while including also the molecule in the system, by increasing subsequently the substrate size, is given in Figure 5. The GPU implementation of the simulation including the molecule took longer than the one for only the metallic substrate and reached quickly one hundred milliseconds per 100 iterations for a small substrate size, but the simulations still ran smoothly in real time. A possible reason for the stagnation of the performance (as compared to the pure gold system) is that the GPU memory was getting filled up, causing the rest of the operations to be stalled [36]. Another source of delay, revealed by a profiling tool that is part of the CUDA development kit, was the kernel launching overhead. Since the threads operate on few data and thus finish their job quickly, this overhead became relatively large and could even dominate the execution scheme in terms of latency.

**Figure 6:**
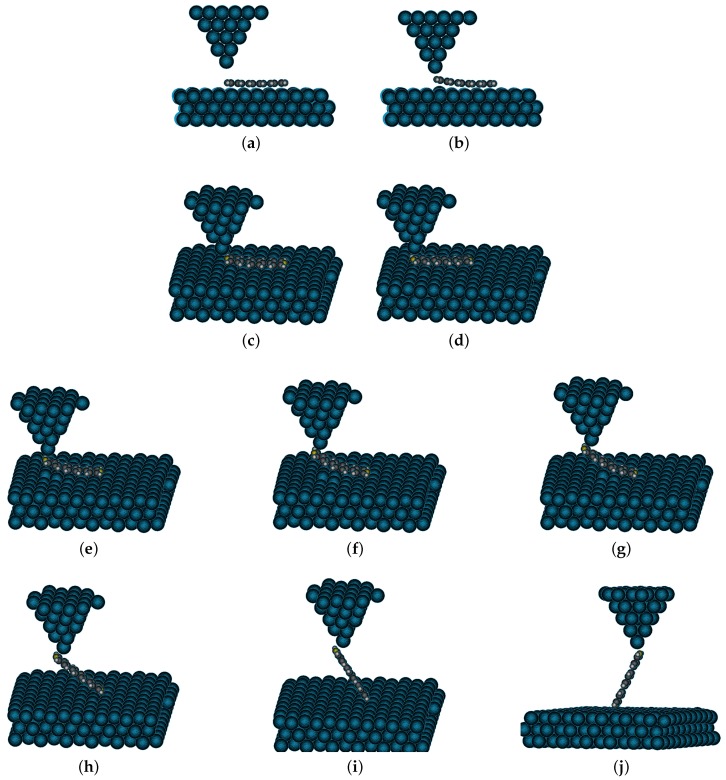
Snapshots for different manipulation operations performed using our GPU implementation of the real-time MD simulation. (**a**) The metallic tip is brought close to the N atom in the 1,4-bis(4-pyridyl)benzene molecule followed by a bond formation at (**b**). (**c**,**d**) The molecule is dragged over the Au(111) surface using the Au tip. After this sequence, (**e**–**j**) shows a lifting operation of the molecule from the metallic substrate.

Figure 6 shows snapshots of the real-time simulation output demonstrating a sequence of images showing dragging and lifting of a single molecule by the tip over a Au(111) substrate. Here, the positions of the gold atoms in contact with the molecule were calculated taking into account both the external input and the relaxation of the molecule. The interaction between the substrate gold atoms was calculated using an embedded atom-potential [24,25], while the tip gold atoms were treated using a harmonic potential (the parameters used in the simulation are given in Appendix Table A1). The lifting of a molecule in experiments was helped by the higher reactivity of the tip apex atoms (as a consequence of the lower coordination number) as compared to the substrate gold atoms. However, as the Morse potential used here for simulating the Au-N bond was a pair potential, we needed to separately enhance the reactivity of the tip as compared to the substrate gold atoms. To this effect, we manually defined a different affinity for the N atom in the molecule towards the tip gold atoms and the surface gold atoms. The manipulations that are shown in Figure 6 were performed with a real-time user-input as described earlier in Section 1. Such user-input came from a 3D motion control system or a joystick, giving x,y,z position signal in real-time to the top layer of the tip atoms in the simulation. It thus demonstrated a successful implementation of the real-time molecular dynamic simulation using the GPU programming.

Although the implementation was successful, the actual physical parameters used in the simulation should be adjusted based on experimental inputs. One such input was obtained by comparing the structure of a molecule when adsorbed on a metallic substrate [37] between the experiment and the simulation. Note that the molecule shown in the simulation was lying flat on the gold surface. In reality, each atom of the molecule (N, C, H) made a different bond (with different bond strengths and bond lengths) with the substrate gold atoms. This means that different parameters (in the Lennard–Jones potential) for different substrate-molecule atom combination have to be used, and these values should be tested against the experiment. For simplicity, we have used the same parameters for all the substrate-molecule atom combinations as given in Appendix (Table A1), which gave a flat molecule structure over the metallic substrate.

## 5. Conclusions and Outlook

We have developed a continuous steering real-time molecular dynamic simulation which can provide real-time feedback while doing a controlled molecular manipulation using low-temperature SPM. We prepared this simulation using CUDA GPGPU programming, which allowed us to compute different parts of the simulation in parallel. Most importantly, the multiple time step problems arising from a large difference in the mass of metallic atoms and their molecular counterparts were solved by a judicious distribution of computations between different thread blocks and thus running many molecule iterations in parallel to a single iteration of the metallic system. All the intramolecular interactions and the metal-molecule interactions were computed while maintaining the real-time performance of the system. This program allows one to track the changes in the molecule conformations and rotations during molecular manipulation. The speed-up is almost two orders of magnitude for a pure gold system, and even the complete system including molecules runs more than an order of magnitude faster than the pure metallic system simulated on a CPU.

The advantage in runtime really pays off: the successful parallel implementation makes the simulation a useful tool for STM experimentation. A system combining a low-temperature STM, a 3D motion control system and a real-time molecular dynamic simulation is useful for realizing a benchmark testing machine for single-molecule electronic transport. From here, different conductance values recorded during the experiment can be attributed to particular metal-molecule-metal configurations suggested by the MD simulation. These configurations provide atomic-scale information about how the molecule is coupled to the metallic leads and can then be used as an input for more detailed electronic transport calculations (DFT, NEGF, etc.), and the values of conductance and coupling strength thus predicted by the theory can be compared hand in hand with the experimental data. As an outlook, the simulation can be further upgraded to include larger 3D molecules and not only planar. This upgrade will require writing the shared memory allocation in a different manner, as more atoms’ data have to be shared among all the thread blocks.

## Figures and Tables

**Table 1:** Device structure-related properties.

CUDA Device Name	GeForce GTX 960
Compute capability	5.2
Floating-point performance	2.413 TFLOPS
GPC count	2
SMMper GPC	4
Cores per SMM	128
Threads per SMM	2048
Max # of threads per block	1024
Max block size	1024 × 1024 × 64
Max grid size	2,147,483,647 × 65,535 × 65,535
Warp size	32 *threads*
Max # active warps per SMM	64 *warps*

**Figure A1:**
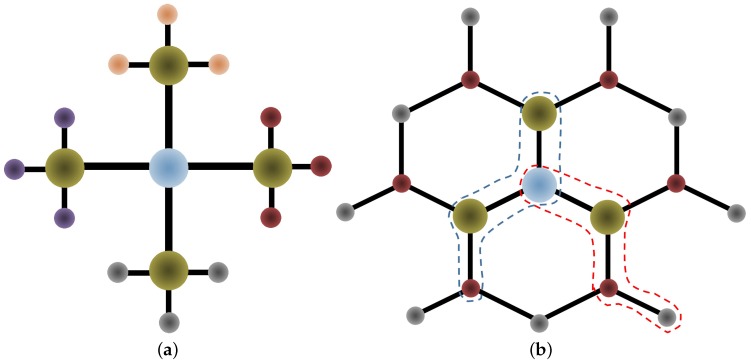
A geometric construction of a molecule with (**a**) n=4 bonds per atom and (**b**) n=3 bonds per atom.

## Abbreviations

The following abbreviations are used in this manuscript:

GPU	Graphics processing unit
GPGPU	General purpose graphics programming unit
CUDA	Compute unified device architecture
API	Application programming interface
CPU	Central processing unit
GPC	Graphics processing cluster
STM	Scanning tunneling microscope
UHV	Ultra-high vacuum
MD	Molecular dynamics
NTS	Native time-scale
FLOPS	Floating point operations per second
DFT	Density functional theory
NEGF	Non-equilibrium Greens function

## Appendix A. Intramolecular Forces

The intramolecular forces consist of bond forces, angular forces and torsional forces. For a molecule, with a maximum of *n* bonds per atom (single, double or triple covalent bonds are all counted as one bond), the maximum number of bond force calculations $n_{b}$ per atom is *n*.

Next, we estimate the maximum number of angular force calculations $n_{a}$ per atom and the maximum number of torsional force calculations $n_{t}$ per atom. For this, consider the illustration in Figure A1a. It shows a simplified geometric construction of a molecule with four bonds per atom. In reality, a molecule with four bonds per atom is more likely to be non-planar, but for the sake of simplicity, we draw it planar structure here. In the picture, the center atom (shown in blue color) has four first neighbor atoms (shown in green color), and each of them has their respective four neighbor atoms, including the central blue atom.

To estimate the maximum number of angular force calculations per atom, we calculate them for the central blue atom. The number of ways in which the central blue atom makes an angular bond with only the first neighbors (green atoms) is equal to the number of ways in which it can choose any two of the green atoms, which is ${}^{4}C_{2}$. In the general case for *n* bonds per atom, it is ${}^{n}C_{2}$, which is n(n−1)/2. Next, the central blue atom can also make angular bonds with one green atom and one of its other neighbors (i.e., purple, grey, orange or red atoms). The number of ways in which this can be done is equal to the number of ways the central blue atom chooses one of the green atoms (i.e., *n* ways) times the number of ways the chosen green atom chooses one of its neighbors excluding the blue atom (i.e., (n−1) ways). This makes the total equal to ${}^{n}C_{2}+n\left(n-1\right)$. Thus, the maximum number of angular forces calculations $n_{a}$ per atom is $\frac{3}{2}n\left(n-1\right)$.

The estimation of the maximum number of torsional force calculations $n_{t}$ per atom is done similarly as the previous calculation. However, the difference is that for torsional forces, we have to make a group of four atoms connected in a chain fashion. For this, we look at Figure A1b. We need to calculate how many groups of four atoms the central blue atom can make. The central blue atom can make an outer group (shown with the red dashed loop) or an inner group (shown with the blue dashed loop) depending on its position in the group. The number of outer groups that the central atom makes is equal to the number of ways in which the central blue atom chooses one of its first green neighbors times the number of ways the chosen green atom further chooses one of its red neighbors times the number of ways the chosen red atom finally chooses one of its grey neighbors. This combines into = n×(n−1)×(n−1).

The number of inner groups (shown with the blue dashed loop in Figure A1) that the central blue atom makes is equal to the number of ways the central blue atom chooses any two of its first neighbor green atoms times the number of ways one of the two chosen green atoms is selected times the number of ways the selected green atom then chooses one of the red neighbor atoms. This combines into ${}^{n}C_{2}$×2×(n−1).

This gives the total torsional groups, and thus, the maximum number of torsional forces calculations $n_{t}$ per atom is ${}^{n}C_{2}2\left(n-1\right)+n{\left(n-1\right)}^{2}$ = $2n{\left(n-1\right)}^{2}$.

## Appendix B. Gold-Molecule Force Calculations

Covalent bonds are usually modeled using harmonic potentials, where the restoring force increases linearly with the bond-length. This simple potential can describe the intermolecular bonds very well and is a good assumption in most situations where the bonds are not stretched enough to break them. However, for studying or simulating the bond formation between the metallic lead (Au) and the anchoring group (N or S), one has to look for more advanced potentials like Morse and Lennard–Jones (LJ) potentials. As the Morse potential decays faster than the LJ potential, we use it to simulate the stronger Au-N bond, while LJ potentials are used to simulate Au-C and Au-H bonds.

### Appendix B.1. Morse Potential: Au-N Bond

The Morse potential is given as:

(A1) $$E=\sum_{i}^{N}\sum_{j\neq i}^{N}D_{e}{\left(1-e^{-\alpha (r_{ij}-r_{\mathrm{eq}})}\right)}^{2}$$

Here, $\alpha =\sqrt{\kappa_{e}/2D_{e}}$ and $\kappa_{e}=\kappa_{\mathrm{Au}-N}$ is the force constant at the minimum of the potential where a harmonic approximation is valid. From here, the *k*-th component (here, *k* can be either *x* or *y* or *z*) of the force on an atom with index *a* can be written as:

(A2) $$\begin{array}{cc} F_{ak} & =-\frac{\partial E}{\partial r_{ij}}\frac{\partial r_{ij}}{\partial k_{a}}\\ & =-\frac{\partial }{\partial r_{ij}}\sum_{i}^{N}\sum_{j\neq i}^{N}D_{e}{\left(1-e^{-\alpha (r_{ij}-r_{\mathrm{eq}})}\right)}^{2}\frac{\partial r_{ij}}{\partial k_{a}}\\ & =-\sum_{i}^{N}\sum_{j\neq i}^{N}2D_{e}\left(1-e^{-\alpha (r_{ij}-r_{\mathrm{eq}})}\right)\alpha e^{-\alpha (r_{ij}-r_{\mathrm{eq}})}\left[\frac{k_{a}-k_{j}}{r_{aj}}\delta_{ia}-\frac{k_{i}-k_{a}}{r_{ia}}\delta_{aj}\right]\\ & =-2\alpha D_{e}\left[\sum_{j\neq a}^{N}e^{-\alpha (r_{aj}-r_{\mathrm{eq}})}\left(1-e^{-\alpha (r_{aj}-r_{\mathrm{eq}})}\right)\frac{k_{a}-k_{j}}{r_{aj}}\right.\\ & \left.-\sum_{i\neq a}^{N}e^{-\alpha (r_{ia}-r_{\mathrm{eq}})}\left(1-e^{-\alpha (r_{ia}-r_{\mathrm{eq}})}\right)\frac{k_{i}-k_{a}}{r_{ia}}\right]\\ & =-4\alpha D_{e}\sum_{i\neq a}^{N}e^{-\alpha (r_{ia}-r_{\mathrm{eq}})}\left(1-e^{-\alpha (r_{ia}-r_{\mathrm{eq}})}\right)\frac{k_{a}-k_{i}}{r_{ia}} \end{array}$$

### Appendix B.2. Lennard–Jones Potential

The LJ potential has the following functional form:

(A3) $$E=4\epsilon \sum_{i}^{N}\sum_{j\neq i}^{N}\left[{\left(\frac{\sigma }{r_{ij}}\right)}^{12}-{\left(\frac{\sigma }{r_{ij}}\right)}^{6}\right]$$

Same as before, the *k*-th component of the force on an atom with index *a* can be written as:

(A4) $$\begin{array}{cc} F_{ak} & =-\frac{\partial E}{\partial r_{ij}}\frac{\partial r_{ij}}{\partial k_{a}}\\ & =-4\epsilon \frac{\partial }{\partial r_{ij}}\sum_{i}^{N}\sum_{j\neq i}^{N}\left[{\left(\frac{\sigma }{r_{ij}}\right)}^{12}-{\left(\frac{\sigma }{r_{ij}}\right)}^{6}\right]\frac{\partial r_{ij}}{\partial k_{a}}\\ & =-4\epsilon \sum_{i}^{N}\sum_{j\neq i}^{N}\left[-12\frac{\sigma^{12}}{{r_{ij}}^{13}}+6\frac{\sigma^{6}}{{r_{ij}}^{7}}\right]\left[\frac{k_{a}-k_{j}}{r_{aj}}\delta_{ia}-\frac{k_{i}-k_{a}}{r_{ia}}\delta_{aj}\right]\\ & =-48\epsilon \sum_{i\neq a}^{N}\left[-2\frac{\sigma^{12}}{{r_{ai}}^{14}}+\frac{\sigma^{6}}{{r_{ai}}^{8}}\right]\left(k_{a}-k_{i}\right) \end{array}$$

The position of the energy minimum and the width and depth of the potential well in both the Morse and the LJ potentials have to be determined using the experiments. For this deformation in the molecular structure on absorption on a metallic surface, its absorption height and absorption energy can be used to fix the unknown parameters. For a proof of principle demonstration of the simulation, we have used the following parameters in our case.

**Table A1:** Parameters used in the simulation.

Parameters	Values	Units
$\kappa_{\mathrm{Au}-N}$ *	325	N/m
$D_{e}$ **	1	eV
$r_{\mathrm{eq}}$	2.5	Å
σ	2.2	Å
ϵ	0.02	eV
$\kappa_{\mathrm{Au}-\mathrm{Au}}$ *	8	N/m

* Value from Rascón-Ramos et al. [38]. ** Value similar to Fournier et al. [39].
